# Functional effects of KCNQ K^+^ channels in airway smooth muscle

**DOI:** 10.3389/fphys.2013.00277

**Published:** 2013-10-07

**Authors:** Alexey I. Evseev, Iurii Semenov, Crystal R. Archer, Jorge L. Medina, Peter H. Dube, Mark S. Shapiro, Robert Brenner

**Affiliations:** ^1^Department of Physiology, University of Texas Health Science Center at San AntonioSan Antonio, TX, USA; ^2^Department of Microbiology, University of Texas Health Science Center at San AntonioSan Antonio, TX, USA

**Keywords:** KCNQ, K_v_7, airway smooth muscle, muscarinic receptors, patch-clamp electrophysiology, voltage-gated potassium channels

## Abstract

KCNQ (K_v_7) channels underlie a voltage-gated K^+^ current best known for control of neuronal excitability, and its inhibition by G_q/11_-coupled, muscarinic signaling. Studies have indicated expression of KCNQ channels in airway smooth muscle (ASM), a tissue that is predominantly regulated by muscarinic receptor signaling. Therefore, we investigated the function of KCNQ channels in rodent ASM and their interplay with G_q/11_-coupled M_3_ muscarinic receptors. Perforated-patch clamp of dissociated ASM cells detected a K^+^ current inhibited by the KCNQ antagonist, XE991, and augmented by the specific agonist, flupirtine. KCNQ channels begin to activate at voltages near resting potentials for ASM cells, and indeed XE991 depolarized resting membrane potentials. Muscarinic receptor activation inhibited KCNQ current weakly (~20%) at concentrations half-maximal for contractions. Thus, we were surprised to see that KCNQ had no affect on membrane voltage or muscle contractility following muscarinic activation. Further, M_3_ receptor-specific antagonist J104129 fumarate alone did not reveal KCNQ effects on muscarinic evoked depolarization or contractility. However, a role for KCNQ channels was revealed when BK-K^+^ channel activities are reduced. While KCNQ channels do control resting potentials, they appear to play a redundant role with BK calcium-activated K^+^ channels during ASM muscarinic signaling. In contrast to effect of antagonist, we observe that KCNQ agonist flupirtine caused a significant hyperpolarization and reduced contraction *in vitro* irrespective of muscarinic activation. Using non-invasive whole animal plethysmography, the clinically approved KCNQ agonist retigabine caused a transient reduction in indexes of airway resistance in both wild type and BK β1 knockout (KO) mice treated with the muscarinic agonist. These findings indicate that KCNQ channels can be recruited via agonists to oppose muscarinic evoked contractions and may be of therapeutic value as bronchodilators.

## Introduction

The control of membrane voltage by K^+^ channels serves as a negative feedback to oppose voltage-dependent calcium influx pathways that contribute to airway smooth muscle (ASM) contraction. K^+^ channel agonists may be useful as bronchodilators for asthma since ASM hyperpolarization by K^+^ channel openers can partly relax ASM (Pelaia et al., 2002). In addition, the most common treatments for asthma, β-adrenergic agonists, apparently confer much of their effects through activation of large conductance Ca^2+^-activated (BK-type) K^+^ channels (Kotlikoff and Kamm, 1996).

Recent studies have uncovered a new role for KCNQ (K_v_7) K^+^ channels in control of contraction of various smooth muscle cell types (Greenwood and Ohya, 2009; Gurney et al., 2010), including guinea pig and human ASM (Brueggemann et al., 2012). Called “M channels” for their depression by stimulation of muscarinic acetylcholine receptors (mAChRs) in neurons, they have an established role in regulation of excitability in nerve and heart (Brown et al., 1997). KCNQ channels are encoded by five genes (KCNQ1–5) and may associate with KCNE accessory β-subunits in a tissue-specific fashion (Soldovieri et al., 2011). Neuronal KCNQ channels are multifariously composed of KCNQ2–5 subunits (Wang et al., 1998; Schroeder et al., 2000; Shah et al., 2002). Recent studies suggest that KCNQ1,4, and 5 are the predominant subunits in smooth muscle (Greenwood and Ohya, 2009). In sympathetic ganglia, KCNQ channels are inhibited by mAChR agonists via depletion of phosphatidylinositol 4,5-bisphosphate (PIP_2_) following cleavage by phospholipase C (PLC); in addition, stimulation of a number of other PLC-coupled receptors also modulate KCNQ channels via multiple intracellular mechanisms (Hernandez et al., 2008). Robust expression of KCNQ channels composed of KCNQ1,4,5 subunits has been documented in vascular smooth muscle, in which they, like BK channels, play a prominent role in controlling contraction Mackie and Byron, 2008; Greenwood and Ohya, 2009). In addition to vascular smooth muscle, KCNQ channels moderate constriction of bladder, myometrium, and gut smooth muscle (Anderson et al., 2009; Greenwood et al., 2009; Jepps et al., 2009; Joshi et al., 2009), implicating KCNQ channels as novel targets for numerous disease states involving smooth muscle (Mackie and Byron, 2008). Expression of KCNQ3 and 5 channels has been suggested in airway epithelia (Greenwood et al., 2009) and ASM (Kakad et al., 2011), suggesting the nascent emergence of an, as yet, under-studied field with high relevance to pulmonary health and disease.

Given the critical role of PLC-coupled M_3_ mAChRs in ASM, it seems logical that acetylcholine (ACh) acts in those cells, at least in part, by modulation of KCNQ channel activity, however, this hypothesis remains to be tested. Moreover, a number of KCNQ openers have been developed that were first targeted as anti-epileptics (Padilla et al., 2009; Wickenden and McNaughton-Smith, 2009; Fritch et al., 2010). Thus, there is the exciting possibility that novel drugs targeting KCNQ channels may provide novel therapeutics for smooth muscle disorders, including asthma.

Here, we address the role of KCNQ channels in control of membrane voltage and contractions of ASM. Whereas KCNQ channels have been shown to moderate muscarinic agonist-evoked contractions of ASM (Brueggemann et al., 2012), their role in the context of muscarinic signaling and their relationship to other K^+^ currents needs further study in that tissue. For example, it is unclear whether M-current inhibition of KCNQ channels is relevant to ASM and how this might affect KCNQ agonists as bronchodilators. Do KCNQ channels have redundant function with the more established ASM K^+^ channel, the BK channel, or do the two types of K^+^ channels work in parallel? In this study, we investigate the role of KCNQ channels in mouse and rat tracheal smooth muscle (TSM). We use KCNQ channel pharmacology to evaluate the functional consequences of KCNQ currents on voltage and contraction of rodent ASM. We also utilize BK channel β1 knockout (KO) mice to understand the complementary role of BK and KCNQ channels in ASM.

## Materials and methods

### Ethical approval

All animal procedures were designed to be as humane as possible, and were reviewed and approved by the University of Texas Health Science Center at San Antonio Institutional Animal Care and Use Committee. These procedures were in accordance with the U.S. National Institutes of Health guidelines.

### Tissue preparations and contraction recordings

The BK channel β_1_ subunit KO mice are congenic by seven generations of inbreeding to the C57BL/6 line of Jackson Labs (strain C57BL/6J) and maintained as homozygous lines. Control animals used in these studies were 2–3 month old C57BL/6J mice strain from Jackson Labs, or 2–3 week old rats (Sprague Dawley) from Charles River Labs. For tracheal constriction studies we used previously published protocols (Semenov et al., 2012). Animals were deeply anesthetized with isoflurane and then sacrificed by cervical dislocation. Trachea were quickly removed and dissected clean of surrounding tissues in ice-cold normal physiological saline solution (PSS). The tracheal tube was cut below the pharynx and above the primary bronchus bifurcation. Two metal wires, attached to a force transducer and micrometer (Radnoti, LLC), were threaded into the lumen of the trachea. The trachea was placed into an organ bath oxygenated by an O_2_-CO_2_ mixture (95% O_2_, 5% CO_2_), at 37°C. Resting tension was continuously readjusted to 10 mN for 1 h and then challenged with 67 mM K^+^ PSS twice or more until reproducible contraction responses were achieved. Subsequent experimental challenges with drugs were normalized to the constriction response to the 67 mM K^+^ PSS solution. In 67 mM K^+^ PSS, the K^+^ reversal potential is depolarized and therefore K^+^ currents are unlikely to play a role in controlling membrane potential and contraction tone. For experiments that involve two muscarinic challenges, we found that the second challenge does not show significant fatigue (*P* = 0.19, *N* = 9 for WT, *P* = 0.18, *N* = 9 for KO, students paired *t*-test). On average, the second challenge shows less than 1% reduction in response from that of first challenge for WT and β1 KO trachea, respectively. Normal PSS used was (mM) 119 NaCl, 4.7 KCl, 2.0 CaCl_2_, 1.0 KH_2_PO_4_, 1.17 MgSO_4_, 18 NaHCO_3_, 0.026 EDTA, 11 glucose, and 12.5 sucrose. The pH of the solution was adjusted to 7.35 by a 95% O_2_ - 5% CO_2_ mixture. The 67 mM K^+^ PSS utilized reduced sodium (56.7 mM NaCl) to maintain proper osmolarity. “Low K^+^” PSS solution was used to measure contractions in relatively hyperpolarized conditions. Low K^+^ PSS consisted of 1.0 mM K^+^ derived from the KH_2_PO_4_ in the PSS solution, with no added KCl. Normal PSS has a total 5.7 mM K^+^ derived from 4.7 mM KCl plus 1.0 mM KH_2_PO_4_. All other ingredients were unchanged.

### Tracheal smooth muscle cell isolation and patch clamp recording

Tracheas were isolated as described above. The dorsal muscle layer was cut away from the hyaline cartilage rings and minced into ~1-mm pieces in Ca^2+^-free HEPES-buffered Krebs solution (140 mM NaCl, 4.7 mM KCl, 1.13 mM MgCl, 10 mM HEPES, 10 mM glucose, pH 7.3). After addition of 2.5 U/ml papain (Worthington), 1 mg/ml BSA fraction V, and 1 mg/ml dithiothreitol, TSM tissue was agitated at 37°C on a shaking platform (250 moves/min) for 20 min. Tissue was washed once with the Ca^2+^-free Krebs solution and digested with 12.5 U/ml of type VII collagenase (Sigma Chemical) for 10 min on a rocking platform at 37°C. Digested pieces of tissue were washed three times in Ca^2+^-free Krebs-BSA solution by centrifugation (750 g for 2 min). The tissue was then triturated up to 5 min to disburse single tracheal myocytes. TSM cells were stored on ice in Ca^2+^-free Krebs-BSA solution and used the same day. A small (50 μ l) aliquot of the solution containing isolated tracheal myocytes was placed in an open 1.0 ml perfusion chamber mounted on the stage of an inverted microscope. The TSM cells were allowed to adhere to the glass bottom of the chamber for 20 min and then were perfused (2 ml/min) with Krebs solution.

Membrane potentials (V_m_) and ionic currents of TSM cells were measured using perforated-patch whole cell recordings. Pipettes were pulled from borosilicate glass capillaries (1B150F-4; World Precision Instruments, Sarasota, FL) using a Flaming/Brown micropipette puller P-97 (Sutter Instruments, Novato, CA) and had resistances of 2–4 MΩ when filled with internal solution and measured in standard bath solution. Membrane current was measured with pipette and membrane capacitance cancellation, sampled at 100 μ s, and filtered at 2.9 kHz using an EPC-10 amplifier, and PATCHMASTER software (HEKA/InstruTech, Port Washington, NY). In all experiments, the perforated-patch method of recording was used with amphotericin B (600 ng/ml) in the pipette (Rae et al., 1991). Amphotericin was prepared as a stock solution as 60 mg/ml in DMSO. The access resistance was typically 10–20 MΩ 5–10 min after seal formation. Cells were placed in 800 μ l perfusion chamber through which solution flowed at 1.5–2 ml/min. Inflow to the chamber was by gravity from several reservoirs, selectable by activation of solenoid valves (Warner Instruments, Hamden, CT). Bath solution exchange was essentially complete by <30 s. Experiments were performed at room temperature.

### Unrestrained whole animal plethysmography

Unrestrained whole animal plethysmography of mice was conducted using the BUXCO system (Buxco Research Systems, Wilmington, NC, USA) according to previous procedures (Lomask, 2006). Eight to twelve week old animals were allowed 20 min to acclimate in the chambers. The challenge was delivered in a final volume of 0.5 ml that was nebulized into 8 chambers for approximately the first 2 min of challenge. Airway resistance, inferred from the parameter *PenH* (Lomask, 2006), was measured before and after introduction of nebulized methacholine (McH, 50 mg/ml) into the plethysmography chambers. After an initial challenge of McH (McH1), followed by a 20 min rest, a second McH challenge (McH2) was given, either alone or together with nebulized retigabine (RTG, 200 μ M) and Penh was measured for 10 min. The Penh baseline values were subtracted from the data.

### Data analysis

Igor 5 (Igor, WaveMetrics Inc.), KaleidaGraph 4.1.1 (Synergy Software), and Excel 2007 (Microsoft Corp.) were used for statistical analyses. Significance was determined with paired or unpaired *t*-test. In the cases when more than two experimental groups were compared, a One-Way ANOVA was applied to determine variability among groups. A *post-hoc* Tukey HSD test was used to compare individual groups. For comparison of two groups with multiple factors, we used a Two-Way ANOVA to distinguish if the groups were different. The effects were deemed significant when a *P* < 0.05 was obtained. The results are expressed as the means ± standard error of means where applicable.

### KCNQ expression studies

#### RT-PCR

**Figure 1:**
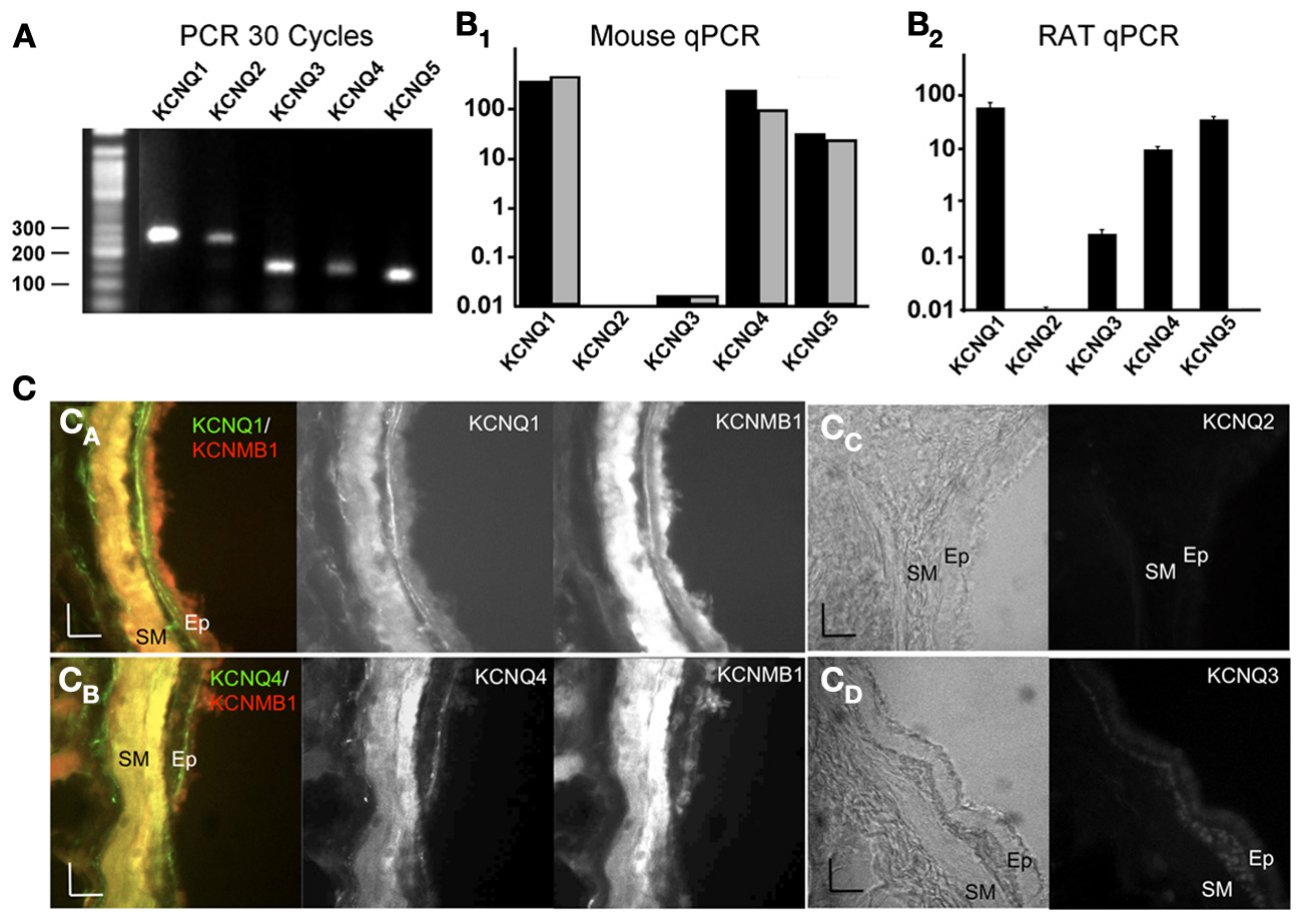
**Expression of KCNQ genes in mouse and rat trachea. (A)** RT-PCR of mouse KCNQ cDNA yields a single expected product size for each KCNQ channel gene. Shown are PCR products following 30 cycles of amplification. The expected amplification products are 243 (mKCNQ1), 226 (mKCNQ2), 136 (mKCNQ3), 127 (mKCNQ4), and 110 (mKCNQ5). The DNA ladder is a 50 bp increment ladder (New England Biolabs N3236). **(B)** Quantitative, real-time RT-PCR from pooled mouse trachea muscle RNA (panel **B_1_**) and rat trachea muscle RNA (*n* = 4, panel **B_2_**) of KCNQ subunit expression normalized to β-actin expression. Gray bars in panel (**B_1_**) are from BK β1 knockout tissues. **(C)** Immunostaining against KCNQ1–4, and BK β1 (KCNMB1) proteins in rat lung sections. Color panels on the left of (**C_A_,C_B_**) are pseudocolor images from double immunofluorescence staining against KCNQ (middle panels) and KCNMB1 (BK β1) (right panels). Panels (**C_C_,C_D_**) include a light image of tissues in the left panels, and immunostaining in right panels. Photographs are taken with the interstitial space to the left, and lumen of the airway to the right. Smooth muscle (SM) and epithelial cell (Ep) layers are indicated. Scale bars are 25 microns.

Tracheal muscle was dissected and pooled from either five wild type or five β1 KO mice. Rat tracheas were analyzed from four individual animals and expression values reflect mean data. The muscle RNA was extracted using Ambion Purelink RNA kit. One microgram of RNA was used for random primer, reverse transcriptase reactions using Applied Biosystems Reverse Transcriptase kit. 1/10 of the cDNA synthesis was used for SYBR green-based PCR amplification of cDNA using the forward and reverse primers listed in Table 1. The thermocycle reactions were conducted in triplicate. Amplification condition was 95°C for 10 min for PCR polymerase activation, and 2 cycle steps of 95°C for 15 s, and 60°C for 60 s for 40 cycles. The specificity of the amplification product was confirmed using melting curve analysis and DNA gel electrophoresis (Figure 1A). The sample cycle thresholds (Ct) were calculated automatically using the Applied Biosystems software to minimize user bias. The mRNA concentrations were calculated using C_t_ levels of target genes normalized to that of β-actin.

**Table 1:** **Mouse (mKCNQ) and rat (rKCNQ) primers utilized for quantitative RT-PCR**.

**Gene**	**Forward primer**	**Reverse primer**	**Product (nt)**
mKCNQ1	GAGGGCAGCACGGTCTATG	GTGGTACACGAAACACTTCCAA	243
rKCNQ1	ACAGGGTGGAAGACAAGGTG	TGGCTACGACTTGTGACCTG	126
mKCNQ2	CAGTACCGAGTTCTTGAGGCT	CTCTGCAATGTAGGGCCTGA	226
rKCNQ2	GTGACTGCGTGGTACATTGG	TGGTTGTCAGGGTGATCAGA	136
mKCNQ3	GAGCCGACAAAGACGGGAC	CTGTACTTGGCGTTGTTCCTC	136
rKCNQ3	GCCCGTTGGCTTAAACAATA	TCTTCTGTCATGGGGTCTCC	111
mKCNQ4	TTGAGCAGTATTCAGCAGGACA	GGACCCTTATCGCCCTTCTC	127
rKCNQ4	GTGGTCTACGCGCACAGTAA	GAGTTGGCATCCTTCTCAGC	107
mKCNQ5	GTCGGCGCAACGTCAAGTA	AACCAAACACAAGGAGAAAAACG	110
rKCNQ5	AATATTTTCGCCACGTCAGC	CTTGCTGTGAGCGTAAACCA	123
mb-actin	AGGTCATCACTATTGGCAACGA	CACTTCATGATGGAATTGAATGTAG	117
rb-actin	AGCCATGTACGTAGCCATCC	ACCCTCATAGATGGGCACAG	115

### Immunostaining

Whole lungs were removed from adult rats. To quickly fix airway passages and associated smooth muscle, an 18 gauge needle was inserted into the trachea and a 4% paraformaldehyde/PBS solution was injected and withdrawn several times (effectively replacing air with fixative). The whole lungs were then fixed overnight in 4% paraformaldehyde/PBS, and washed in 20% sucrose/PBS for a second day. Frozen sections were cut in a sliding microtome at 25 micron thickness and immunostained using the free-floating section technique. Primary antibodies used were Neuromab monoclonal antibodies against KCNQ1 (N37/A10, 0.6 μg/ml), KCNQ2 (N26A/23, 0.6 μg/ml), and KCNQ4 (N43/6, 0.6 μg/ml) and a non-commercial antibody against KCNQ3 (Guinea Pig Anti-N terminal KCNQ3, a gift from Ed Cooper, Baylor College of Medicine) that has been used successfully in the past (Klinger et al., 2011). These antibodies give only specific signals on transiently transfected COS cells as assayed by western blots and/or immunostaining (http://neuromab.ucdavis.edu/catalog.cfm). Guinea pig anti-KCNQ3 (1:1000) antibodies have been successfully utilized for staining of brain sections (Klinger et al., 2011). We did not find antibodies that gave a specific signal for KCNQ5 protein, and therefore immunostaining for KCNQ5 was not performed. Anti-BK channel KCNMB1 (β1 subunit) was using an affinity-purified rabbit polyclonal (1:2000, Pierce Antibody, A1-924). Antibody concentrations used were those with highest dilution that gave a specific signal as compared to no primary antibody controls. Immunostaining and washes were accomplished with 0.2% Tween-20, PBS, and 10% goat serum as blocking agent. Secondary antibodies were at 1:2000 dilution. Images were obtained using an epifluorescent microscope using a 20X 0.5 NA fluor objective and 5 s exposure for all sections including negative controls.

## Results

### Airway smooth muscle predominately express KCNQ1, 4, and 5 subunits

Previously, KCNQ channels were studied in guinea pig ASM and human lung slices (Brueggemann et al., 2012). We first determined if rodents (rats and mice) could also be used as models for KCNQ channel function in ASM, and wished to understand effects of KCNQ channels on resting potentials and muscarinic-evoked contractions *in vitro* and *in vivo*. We assayed KCNQ mRNA expression using quantitative PCR from dissected tracheal muscle. Figure 1A shows that subunit-specific primers specifically detected individual KCNQ subtypes. Real-time, quantitative PCR indicate relative expression levels of KCNQ mRNAs from pooled mouse tissues appear very similar to that previously observed in human airway (Brueggemann et al., 2012) in which KCNQ1 is the predominant subtype followed by KCNQ4 and weak expression of KCNQ5 (expression relative to β-actin: KCNQ1, 366e^−6^, KCNQ4, 243e^−6^, KCNQ5, 32e^−6^, Figure 1B_1_, black bars). Like mice, rat trachea (*n* = 4) show greatest expression of KCNQ1. However, KCNQ5 is somewhat greater than KCNQ4 expression (Figure 1B_2_). In rat, we also observed low levels of KCNQ3 expression that is relatively absent in mice (Figure 1B_2_). We also assayed KCNQ changes in BK channel β1 KO mice, which were used in experiments described later in this study (Figures 5–6). BK channel β1 gene KO mice displayed similar expression levels of KCNQ1–5 as wild type mice (Figure 1B_1_, gray plots) indicating that BK channel β1 KO mice do not compensate with changes in KCNQ channel expression.

Immunostaining of rat lung sections was used to corroborate expression of KCNQ channels in ASM cells. Lung sections of lower airway were immunostained with KCNQ subunit-specific antibodies (see Methods). We observed relatively strong staining of KCNQ1 and KCNQ4 proteins in the smooth muscle layer lining the airway (Fluorescein signal, Figures 1C_A_,C_B_, middle panels) that was absent with no-primary antibody controls (not shown) or with KCNQ2 and KCNQ3 primary antibodies (Figures 1C_C_,C_D_). This appeared to overlap with BK KCNMB1 (β1) protein expression that is documented to express in ASM (Brenner et al., 2000; Semenov et al., 2006) (Texas Red signal, Figures 1C_A_,C_B_, right panels). Indeed, the Fluorescein KCNQ (green) and Texas Red BK β1 (red) signals show a predominant overlap (yellow) in the smooth muscle layer of the airway (Figures 1C_A_,C_B_, left panels). Consistent with RT-PCR, KCNQ2 was not detected in lung (Figure 1C_C_), and KCNQ3 immunostaining showed weak expression, albeit in epithelial cell layers of the airway (Figure 1C_D_). Unfortunately, we could not obtain an antibody that specifically detects KCNQ5 protein in airway.

### The KCNQ current in ASM cells is sensitive to KCNQ-specific drugs and modestly depressed by muscarinic agonists

**Figure 2:**
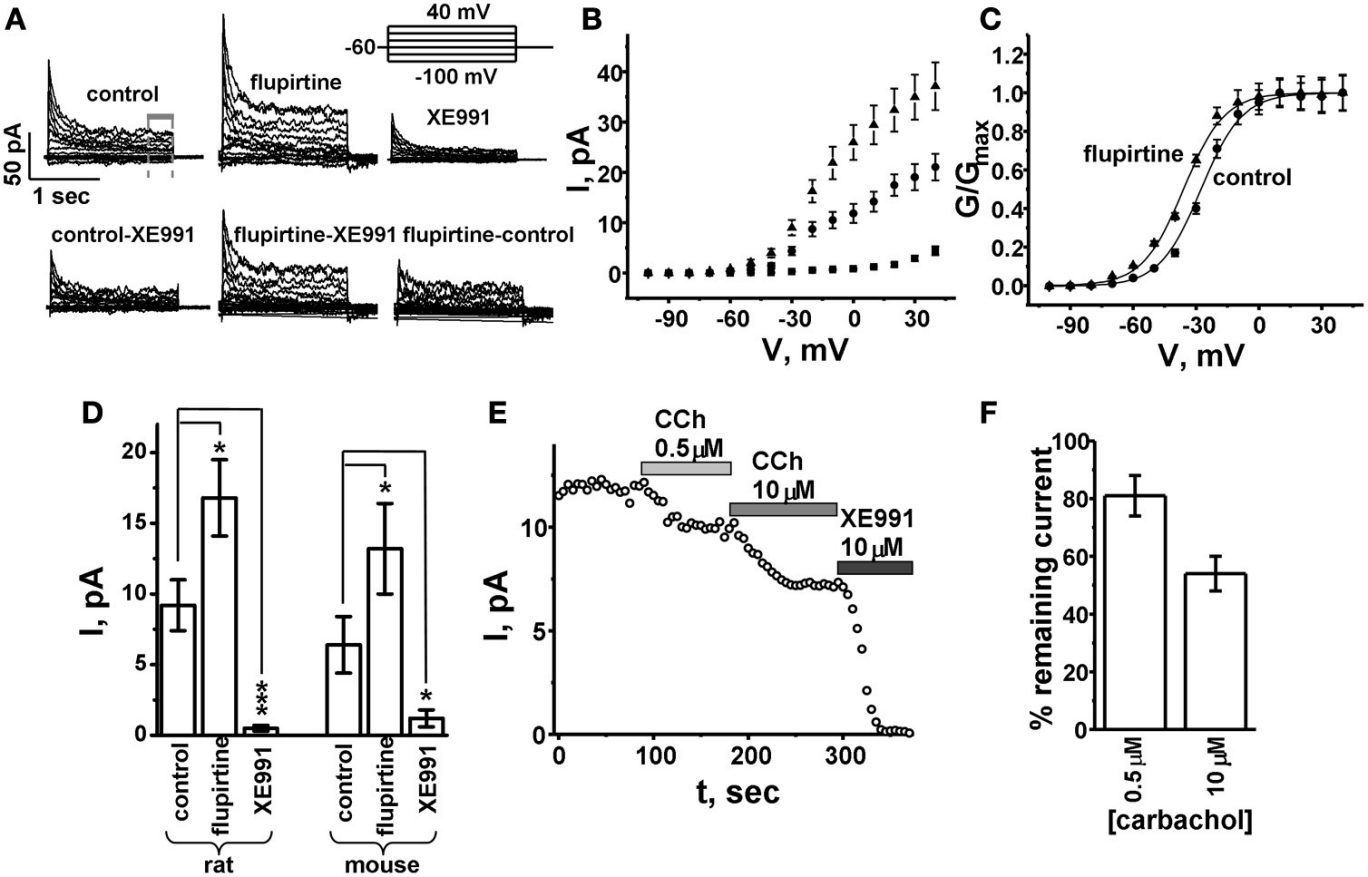
**Isolation of KCNQ currents from tracheal smooth muscle cells. (A)** Representative whole-cell currents from acutely dissociated tracheal smooth muscle cells. All recordings were made with 1 μM paxilline in the bath to block BK K^+^ currents. The KCNQ component was quantified from the final 500 ms of a 2 s voltage command (for panels **B–F**). Bottom panels show the subtracted currents as indicated. **(B)** Average sustained current-voltage relationship. **(C)** Conductance-voltage relationship. **(D)** Average sustained current amplitudes measured at −20 mV holding potential. **(E)** Representative sustained current amplitudes (at −20 mV) with sequential application of 0.5 and 10 μ M carbachol, and 10 μ M KCNQ antagonist XE991. **(F)** Average % remaining current from experiments as in panel **(E)**. ^*^p < 0.05; ^***^p < 0.0001, evaluated by unpaired, two-tailed *t*-test.

KCNQ currents were assayed in acutely dissociated tracheal muscle cells using perforated, whole-cell patch clamp recordings. The bath solution included the BK channel blocker paxilline (1 μ M) to enrich for the KCNQ current fraction. To estimate KCNQ currents in these experiments, we used two approaches. The first exploits the much slower kinetics of KCNQ current activation, compared to other K_v_ channels. The second approach was pharmacological, using KCNQ channel openers and blockers. Thus, we used long (2 s) voltage commands and quantified the KCNQ current as that present during the last 400 ms of each sweep (Figure 2A, control panel), a paradigm similar to that used previously in the study of KCNQ channels in ASM (Brueggemann et al., 2012). Indeed, currents in response to voltages positive to −50 mV displayed an early inactivating component, and a sustained component that is dominated by current from KCNQ channels (Figure 2A). This conclusion was strengthened using paired recordings in the presence or absence of the KCNQ opener, flupirtine and blocker, XE991 (Zaczek et al., 1998). At potentials positive to −60 mV, flupertine (10 μ M) increased the late component of the currents, whereas XE991 (10 μ M) nearly abolished it. Representative currents from rat ASM cells are shown in Figure 2A, pooled current-voltage data in Figure 2B, and pooled activation curves in Figure 2C. The current-voltage relations indicate that the late component is eliminated by XE991 (Figure 2B), consistent with KCNQ channels underlying the majority of the sustained voltage-dependent K^+^ current (with BK channels blocked). Consistent with previous work, flupirtine roughly doubled the KCNQ current at potentials positive to −40 mV (Figures 2B,D), and shifted the conductance/voltage relation to more hyperpolarized potentials (Figure 2C) (Wickenden et al., 2000). We approximated the voltage-dependence of KCNQ current activation by fitting the *G*/*G*_max_ vs. voltage relations to Boltzmann functions, in which the maximum and minimum were constrained to unity and zero, respectively. In control, the V_1/2_ and slope factor were −28 ± 1.2 mV and 9.9 ± 0.3 mV (*n* = 26), and with flupirtine, they were −36 ± 1.4 mV and 9.1 ± 0.3 mV (*n* = 26), respectively, consistent with the biophysical and pharmacological behavior of KCNQ channels. Since the qRT-PCR data indicated subtle differences in the expression profile of KCNQ1–5 subunits across species, we also investigated KCNQ currents in mouse ASM, and saw a similar effect of flupirtine and XE991 on the currents attributed to KCNQ channels (Figure 2D). In rat ASM cells, the persistent current at −20 mV was 9.2 ± 1.8 pA (*n* = 26), which was increased to 16.8 ± 2.7 pA (*n* = 26) by flupirtine, but reduced to 0.5 ± 0.2 pA (*n* = 23) by XE991. In mouse ASM cells, the values were 6.4 ± 2.0 pA (*n* = 7), 13.2 ± 3.2 (*n* = 5) and 1.2 ± 0.6 pA (*n* = 4), respectively. Similarly, flupirtine shifted the V_1/2_ from −26 ± 2 mV, slope factor 9 ± 0.5 (*n* = 7) to −36 ± 1.7 mV, slope factor 8.9 ± 0.6 (*n* = 7). Thus, both rat and mouse ASM cells display KCNQ current of similar current amplitudes and pharmacological sensitivities.

In neurons, KCNQ2/3 channels are strongly inhibited by G_q_-coupled receptors that activate PLC, inducing hydrolysis of PIP_2_ (Hernandez et al., 2008). In ASM, partial inhibition of KCNQ currents (~50% in guinea pig) by ASM constrictors such as histamine and muscarinic agonists has been reported (Brueggemann et al., 2012). Since ACh, acting on muscarinic acetylcholine receptors (mACh), is the predominant stimulus for contraction of ASM, it is of interest to know if the role of KCNQ channels in control of airway tone is dependent on the extent of mAChR stimulation. We used carbachol (CCh), the classically-used muscarinic agonist in such airway studies, as an agonist. Under similar perforated-patch clamp conditions as above, we tested the inhibition produced by CCh at 0.5 μ M and 10 μ M, corresponding to concentrations that produce half-maximal, or near maximal, contraction of trachea, respectively. We observed a relatively small inhibition of KCNQ current at 0.5 μ M CCh, and one substantially larger at 10 μ M (Figure 2E). At 0.5 μ M and 10 μ M CCh, the KCNQ current remaining was 81 ± 7% (*n* = 5), and 54 ± 6% (*n* = 5), respectively (Figure 2F). These data indicate that KCNQ channels are weakly inhibited by muscarinic agonist, and may participate in control of membrane potential.

### KCNQ channel openers oppose muscarinic-induced depolarization of ASM cells

**Figure 3:**
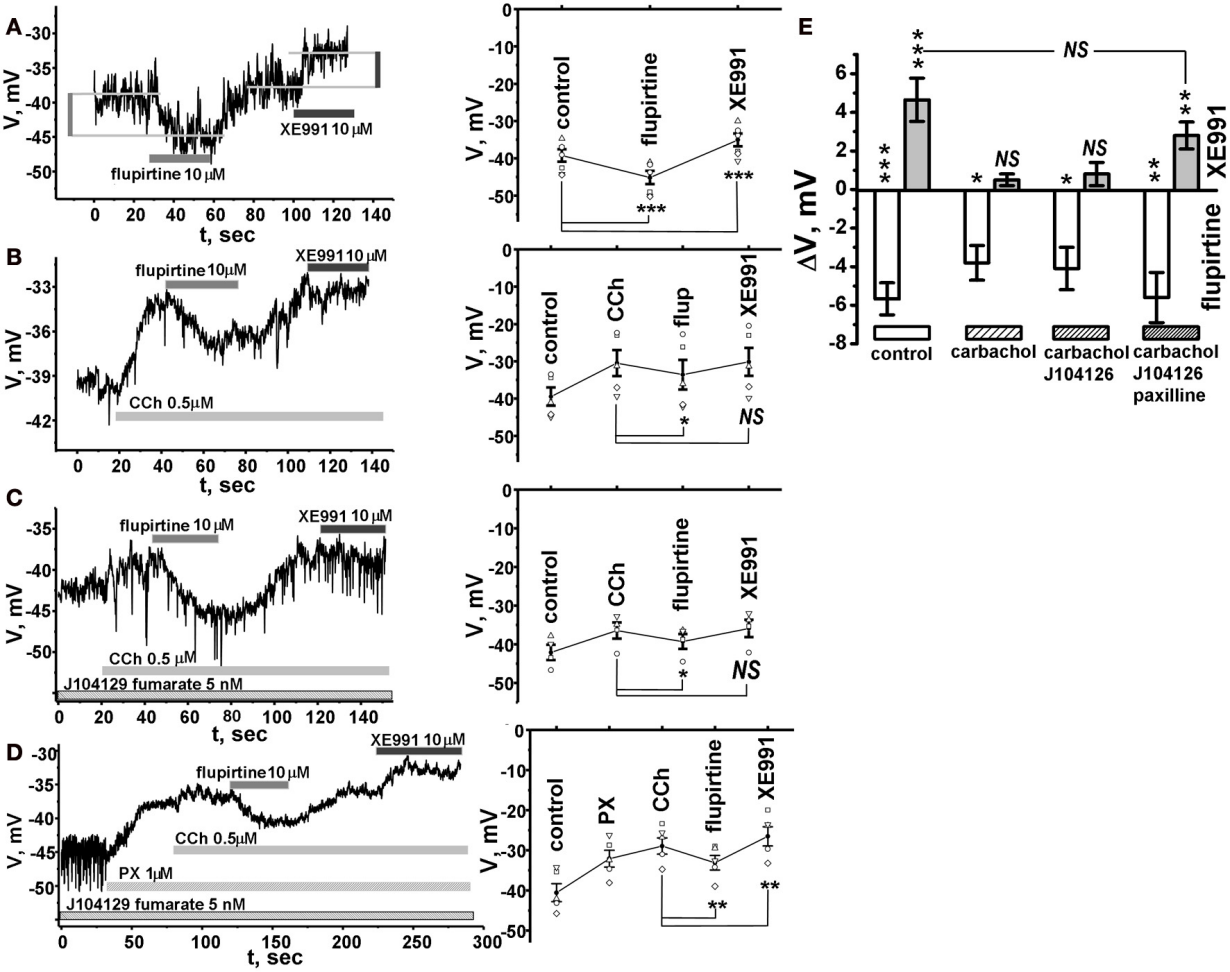
**KCNQ channels affect airway smooth muscle membrane potentials**. Membrane potentials were measured in perforated patch, current-clamp mode. For **(A–D)**, left panels show representative experiments, right panels show average voltage. Statistical significant differences (paired *t*-test) from control **(A)** or carbachol (0.5 μ M) treatment **(B–D)**, is indicated with asterisks. **(A)** Effects of KCNQ agonist flupirtine and antagonist XE991 on membrane potentials at rest (absence of carbachol). Average control −39.2 ± 1.7, flupirtine −45.1 ± 1.8, XE991 −35 ± 1.7. *N* = 6. **(B)** Effects of KCNQ agonist flupirtine and antagonist XE991 on membrane potentials with a cell pre-treated with carbachol. Average control −39.5 ± 2.4, carbachol −30.4 ± 3.4, flupertine −33.6 ± 3.9, XE991 −30.1 ± 3.7. *N* = 5. **(C)** Effects of flupirtine and XE991 on membrane potential in cells pre-treated with M3 receptor antagonist J104129 fumarate and carbachol. Average control −42 ± 1.9, carbachol −37.8 ± 2.1, flupirtine −41 ± 1.9, XE991 −36.6 ± 2.2. *N* = 4. **(D)** Effects of flupirtine and XE991 on membrane potential with a cell pre-treated with M3 receptor antagonist J104129 fumarate (5 nM), BK channel antagonist (1 μ M paxilline) and muscarinic agent (0.5 μ M carbachol). Average control −40.6 ± 2.3, paxilline −32 ± 2.1, carbachol −28.9 ± 2.0, flupirtine −33.1 ± 1.8, XE991 −26.5 ± 2.4. *N* = 5. **(E)** Average change in voltage (ΔV) due to flupertine (white) or XE991 treatment (gray) from control **(A)** or carbachol treatment **(B–D)** measured in **(A–D)**. Changes in voltage for flupirtine and XE991, respectively, are as follows; control: −5.7 ± 0.8, +4.7 ± 1.1; carbachol: −3.8 ± 1, +0.4 ± 0.3, J104129 fumarate and carbachol: −5.1 ± 1.1, +0.5 ± 0.3; J104129 fumarate, J104129 fumarate, paxilline, and carbachol: −5.6 ± 1.2, +2.8 ± 0.7. ^*^p < 0.05; ^**^p < 0.001; ^***^p < 0.0001. Significant differences, indicated with asterisk, were determined using a one-sample *t*-test. NS indicates no significant difference.

Since KCNQ channels are known to control membrane potentials in both nerve and other smooth muscle types, we investigated if they similarly control ASM membrane voltage, using flupirtine and XE991 application while voltages were being measured under perforated-patch, whole-cell current clamp of single tracheal cells. In contrast to the voltage-clamp studies above, these measurements were made in the absence of BK channel blockers to evaluate the physiological contribution of KCNQ channels to control of membrane voltage. As such, voltage oscillations that are reflective of BK channel-mediated spontaneous hyperpolarizations (Zhuge et al., 2010) become apparent in the voltage tracings. A representative experiment is shown in Figure 3A, in which flupirtine (10 μ M) induced a significant hyperpolarization, whereas XE991 (10 μ M) depolarized the cell. Such data are summarized in Figure 3E, in which flupirtine hyperpolarized the cells by 5.7 ± 0.8 mV (*n* = 6) and XE991 depolarized them by 4.7 ± 1.1 mV, relative to control (*n* = 6). The significant effect of flupirtine and XE991 is consistent with the significant KCNQ channel conductance (Figure 2C) starting at the approx. −40 mV resting voltage observed in these cells (Figure 3A, right panel).

We next tested whether the KCNQ channels also affect membrane voltage under mAChR stimulation. Given that KCNQ channel currents are largely active in the presence of CCh at its EC_50_ value for inducing contractions (~80%, Figure 2F), we were surprised to see that XE991 had no significant effect on membrane voltage in the presence of 0.5 μ M CCh (Figure 3B), although flupirtine still induced a significant hyperpolarization. Such data are summarized in Figure 3E, in which flupirtine hyperpolarized the cells by 3.8 ± 1.0 mV (*n* = 5) whereas there was no significant depolarization induced by XE991 in the presence of CCh. This suggests that although KCNQ channels control resting voltage, they may not be necessary for membrane repolarization during muscarinic-evoked contractions.

Parasympathetic release of ACh, and M_2_ and M_3_ mAChR stimulation is the predominant mechanism mediating contraction of airway (Struckmann et al., 2003). Activation of G_q_-coupled M_3_ mAChRs and G_*i*_-coupled M_2_ mAChRs both can evoke tracheal contractions in the absence of the other (Stengel et al., 2002; Semenov et al., 2011). We hypothesized that the lack of effect of KCNQ channels on membrane voltage during muscarinic stimulation could either be due to G_q_-coupled muscarinic inhibition of the channels, or alternatively, due to functional redundancies with other K^+^ channels, such as BK calcium-activated K^+^ channels that regulate membrane voltage during muscarinic-evoked contraction. To address the former, we included the M_3_ receptor antagonist J104129 Fumarate (5 nM) (Mitsuya et al., 1999) to measure effects of XE991 on membrane voltage independent of M_3_ receptor activation. In the presence of the M_3_ antagonist, we again saw no significant effect of XE991 on membrane voltage (Figure 3C, summarized in Figure 3E). To address the latter possibility, we repeated these experiments and included the BK channel blocker paxilline (1 μ M). Under these conditions, XE991 indeed significantly depolarized membrane voltage during muscarinic stimulation (Figure 3D, summarized in 3E). On average, the XE991 depolarization (2.8 ± 0.7, Figures 3D,E) with BK channel block was somewhat smaller than XE991 depolarization in the absence of muscarinic activation (4.7 ± 1.1, Figures 3A,E). However, we did not see a significant difference between the two (*P* = 0.21, unpaired *t*-test). These results are consistent with KCNQ channels being required for control of membrane voltage at rest, but having a redundant role with BK channels during muscarinic signaling. On the other hand, the KCNQ agonist, flupirtine, hyperpolarized the membrane voltage under all circumstances (Figure 3E) indicating that such openers can recruit KCNQ channels to oppose muscarinic-evoked depolarization.

### KCNQ channel blockers do not increase muscarinic-induced contraction of tracheal rings, but KCNQ openers reduce it

**Figure 4:**
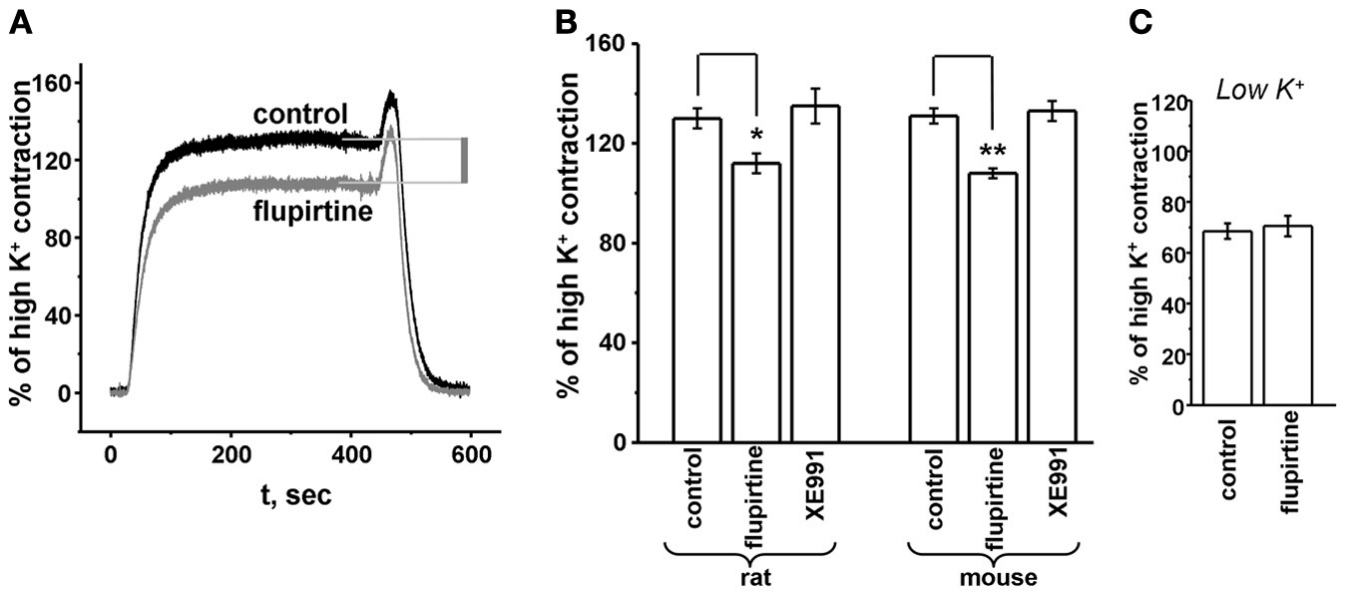
**KCNQ channel opener but not antagonist affect airway smooth muscle contractility. (A)** Representative rat trachea contraction evoked by 0.5 μ M carbachol alone (control) or in the presence of 10 μ M flupirtine. **(B)** Summary tension values for rat and mouse tracheas treated with 0.5 μ M carbachol and 10 μ M flupirtine or 1 μ M XE991. **(C)** Effect of flupirtine on 0.5 μ M carbachol contraction is occluded by a hyperpolarizing, low K^+^ bath solution. Contractions were conducted in mouse tissues. ^*^p < 0.05; ^**^p < 0.001, evaluated by unpaired, two-tailed *t*-test.

Previously, others have shown that KCNQ channel antagonists constrict airways in human lung slices (Brueggemann et al., 2012). Here we investigated the effects of KCNQ channels on rodent airway contractility using isometric tension measurements of tracheal rings. As above, we used flupirtine and XE991 to probe the role of KCNQ channels in CCh-induced contractility. Using 0.5 μ M CCh to elicit tracheal ring contractions, we observed a moderate relaxation (~20%) with flupirtine (Figure 4). Similar to the studies above on voltage (Figure 3), XE991 had no effect on contractility during muscarinic activation (Figures 4A,B). Very similar effects were also seen in mouse trachea (Figure 4B). For rat trachea, the percent of high-K^+^ contraction was 130 ± 4% (*n* = 12), 112 ± 4% (*n* = 12, *p* < 0.005), and 135 ± 7% (*n* = 12) in control, flupertine and XE991, respectively. For mouse trachea, the percent of high-K^+^ contraction was 131 ± 3% (*n* = 3), 108 ± 2% (*n* = 3, *p* < 0.001), and 133 ± 4% (*n* = 3) in control, flupertine and XE991, respectively. The significant effect of the KCNQ-channel opener for both rat and mouse trachea suggests that at potentials where both KCNQ K^+^ and L-type voltage-gated calcium channels (VGCCs) channels are activated, M-channel openers can moderate ASM contraction. As a further test, when we used a low (1 mM) K^+^ external solution to hyperpolarize trachea membrane voltage negative to the voltage threshold for activation of L-type Ca^2+^ channels, there was no effect of flupirtine on the CCh-induced contraction (Figure 4C). In control, the percent of high-K^+^ contractions was 69 ± 3% (*n* = 3), and in flupirtine, it was 71 ± 4% (*n* = 3). This is consistent with a specific effect of flupirtine on membrane voltage via KCNQ channels. In contrast to the effect of flupirtine, XE991 had no effect on CCh-induced contractions (Figure 4B), suggesting that whereas KCNQ channel blockade does not increase contractions, augmenting KCNQ currents moderates CCh-induced ASM contractions.

**Figure 5:**
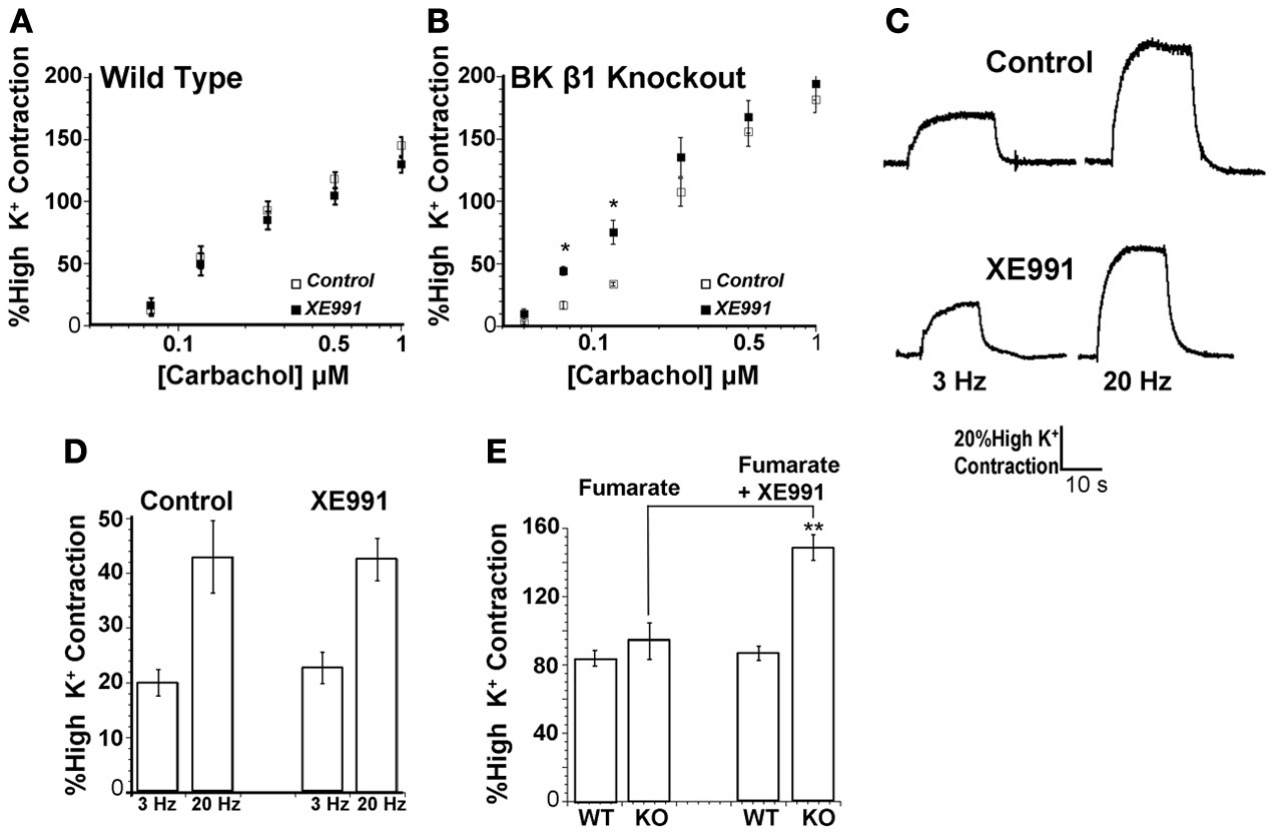
**Effect of KCNQ antagonist is apparent when BK K^+^ channels function is perturbed and M3 receptor activation is blocked**. Effect of KCNQ antagonist XE991 assayed in **(A)** wild type mice and **(B)**. BK channel β1 knockout mice. **(C)** Contractions evoked by electric field stimulation in wild type mice. **(D)** Summary of **(C)**. **(E)** Summary of contractions evoked by 0.5 μ M carbachol and M3 muscarinic acetylcholine receptor antagonist J104129 fumarate (5 nM) alone, or with KCNQ antagonist XE991 (1 μ M). ^*^p < 0.05; ^**^p < 0.001, evaluated by unpaired, two-tailed *t*-test.

The lack of effect of XE991 on CCh-induced contraction may be explained by a robust BK channel current that is sufficient to limit depolarization. To test this hypothesis, we compared the effect of XE991 on contractility of WT mice to mice with a genetically ablated BK channel β1 subunit. β1 KO mice have increased tracheal constrictions due to reduced activation of BK channels (Semenov et al., 2006, 2011). Tracheal contraction was evaluated over a range of CCh concentrations, in the presence or absence of XE991. Consistent with the data in Figure 4 we found that XE991 did not affect CCh-induced trachea contractions in WT mice over the entire range of [CCh] (Figure 5A). However, when BK channel function was perturbed by KO of the BK β1 subunit, we indeed found that KCNQ channel blockade caused increased CCh-evoked contractions that was most pronounced at modest CCh concentrations (Figure 5B). To provide a more physiological paradigm of muscarinic stimulation, we used electrical field stimulation of our intact nerve/trachea preparation, at both moderate and high stimulation rates that should corresponding to modest, or high, [CCh] in Figure 5A. Contractions, measured at stimulation rates of 3 Hz or 20 Hz were compared in the presence or absence of XE991. However, as in Figure 5A, blockade of KCNQ channels did not alter the responses to either high or low stimulation, or the ratio between them (Figure 5C). Such data are summarized in Figure 5D. In control, the percent of high-K^+^ contraction was 20 ± 2.4% and 43.0 ± 6.6% (*n* = 5) at 3 Hz and 20 Hz, respectively, and in the presence of XE991, it was 22.7 ± 2.9% and 42.5 ± 3.9% (*n* = 5), respectively.

### Perturbation of BK channel function allows the KCNQ current to assume a larger role in opposing ASM contractions

The lack of effect of KCNQ blockade on trachea contractility (Figure 4) is consistent with its lack of effect on membrane potential during muscarinic signaling (Figure 3). We investigated this further using the M_3_ receptor antagonist J104129 fumarate (5 nM) and BK channel β1 KO mice to understand how muscarinic G_q_-coupled signaling or redundancies with BK channels, respectively affect the role of KCNQ channels in ASM. We performed these tests at 0.5 μ M CCh, a concentration that produces half-maximal contractions, and at which there was no effect of XE991 both in WT and KO trachea (Figures 5A,B). J104129 fumarate itself reduced the average contractile response to 0.5 μ M CCh by about 30 and 45% in WT and KO trachea, respectively, consistent with M_3_ receptor-mediated Ca^2+^ release of intracellular stores playing a large role in muscarinic-induced contractions of ASM (Figure 5E). Interestingly, whereas XE991 had no effect on J104129 fumarate-treated muscarinic contraction of trachea from wild type mice, the KCNQ channel blocker potentiated the response to CCh by some 1.6-fold in trachea of β1 KO mice (Figure 5E). For WT trachea, the percent of high-K^+^ contraction was 84 ± 4.7% and 87 ± 4.3% (*n* = 8) in the presence or absence of XE991, respectively, whereas in KO trachea, they were 94 ± 10.8% and 149 ± 4.9% (*n* = 7, *p* < 0.001), respectively. These results suggest that when BK channels are fully active, KCNQ channels may be functionally redundant in limiting contractions from membrane depolarization. However, when BK channel function is perturbed, as when the β1 subunit is lacking, KCNQ channels are essential in limiting depolarizations, which likely increase airway contraction via influx of Ca^2+^ through L-type VGCCs. These data further indicate that the lack of significant effect of XE991 at higher CCh concentrations in the KO trachea (Figure 5B) is probably due to KCNQ channels already being inhibited by stimulation of G_q/11_-coupled M_3_ muscarinic receptors (as in Figure 2F). Thus, when that PLC-linked mechanism of KCNQ current depression is occluded by receptor blockade, a very significant effect of blocking KCNQ channels is revealed. In the discussion, we elaborate on the significance of these findings, in the context of asthmatic syndromes associated with altered expression of BK β1 subunit expression.

### KCNQ channel openers retard muscarinic-induced airway constriction in live mice

**Figure 6:**
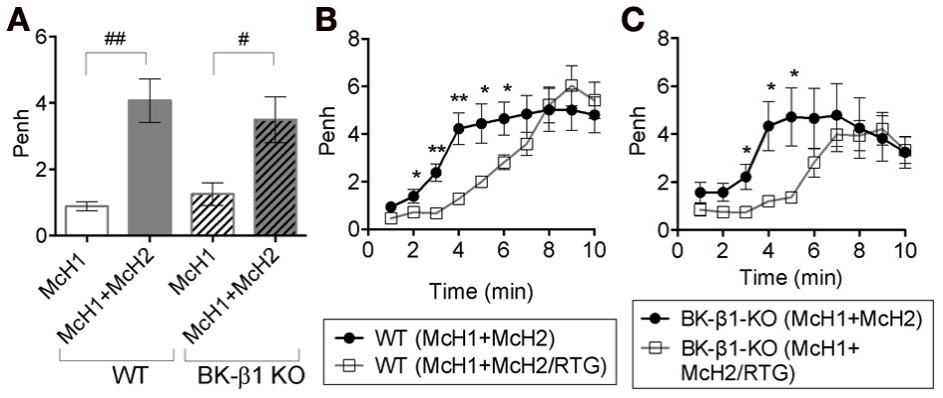
**Retigabine transiently reduces bronchoconstriction by methacholine**. Penh was measured in conscious wild type (WT) and BK-β1 knockout (BK-β1 KO) mice. Measurements were before and after introduction of nebulized methacholine (McH, 50 mg/ml) into the plethysmography chambers. After an initial challenge of McH (McH1), followed by a 20 min rest, a second McH challenge (McH2) was given, either alone or together with nebulized retigabine (RTG, 200 uM) and Penh was measured for 10 min. The Penh baseline values were subtracted from the data. **(A)** The second dose of methacholine significantly increases airway resistance. Penh during the first McH challenge was averaged over 10 min and compared with the 10 min Penh average of the second challenge of McH (McH2). **(B,C)** Penh is plotted for each minute average of the second McH challenge, either alone or together with RTG, for either WT **(B)** or BK-β1 KO **(C)** mice. ^#^*p* < 0.05; ^##^*p* < 0.001, evaluated by unpaired *t*-test corrected for multiple comparisons by Holm Sidak method. ^*^p < 0.05; ^**^p < 0.001, evaluated by paired, two-tailed *t*-tests.

We wished to further investigate the *in vivo* role of KCNQ channels in pulmonary function. We used non-invasive whole animal plethysmography. This technique reports measurements of airway function during normal, passive breathing. Plethysmography does not provide a direct measure of airway resistance, but rather provides an estimate of an enhanced pause (PenH) in breathing mechanics that is correlated to increases of airway resistance (Lomask, 2006). Because airway resistance has a cumulative effect upon repeated muscarinic stimulation, we tested the effects of the clinically approved KCNQ agonist retigabine (RTG, 200 μ M) when it was combined with a second challenge of methacholine (50 mg/ml). PenH was monitored for 10 min following the second challenge with methacholine, either alone or when combined with RTG. Both WT and KO mice displayed a substantially increased PenH value upon the second challenge of methacholine, compared to the first (compare McH1 vs. McH1 + McH2 in Figure 6A, 10 min average response). We examined the PenH response on a temporal basis and calculated the individual 1 min average time-points following the second McH2 or McH2/RTG challenges, as plotted in Figures 6B,C. The data show RTG to significantly reduce the PenH value in both wild type and β1 KO mice during the early 2–6 min of the second challenge. The difference is most significant at early timepoints, with decreased effect of RTG during the later 6–10 min time window. Thus, the effect of KCNQ activation is most significant in the initial 6 min after muscarinic stimulation, whereas its role is less during sustained contractions. These results are consistent with the *in vitro* tracheal studies showing that KCNQ channel activation reduces muscle contraction during muscarinic stimulation (Figure 4).

## Discussion

A major observation of this study is that KCNQ channels control resting membrane potential of ASM, which is consistent with their activation at relatively negative (>−40 mV) membrane potentials. However, muscarinic activation eliminated the effect of KCNQ blockade on membrane potential. While KCNQ agonist flupirtine relaxed muscarinic-evoked contractions, KCNQ blockade did not enhance it. This suggests that, although KCNQ channels can be pharmacologically recruited to relax airway, they do not contribute to relaxation during muscarinic-evoked contractions. In part, one might expect that the mechanism would be due to inhibition by signaling downstream of muscarinic receptor activation and that the muscarinic-evoked depolarization of ASM could be due to muscarinic inhibition of KCNQ channels. However, voltage clamp recordings indicate muscarinic agonist only weakly inhibits KCNQ channels (~19% inhibition at 0.5 μ M CCh, 46% at 10 μ M CCh (Figure 2F), approximate EC_50_ and maximum concentrations for contraction, respectively). This contrasts with sympathetic neurons that show 80–90% KCNQ current inhibition at 10 μ M muscarinic agonist (Beech et al., 1991; Bernheim et al., 1992). Further, blocking M_3_ receptors alone did not uncover control by KCNQ channels (XE991) on voltage or contractions during muscarinic signaling. Rather, the data indicate that functional redundancy with BK channels reduces the contribution of KCNQ channels during ACh-evoked contractions. At low muscarinic agonist concentrations, the BK β1 KO was sufficient to reveal effects of XE991. At higher muscarinic agonist concentrations the combination of β1 KO and M_3_ receptor blockade revealed a significant effect of KCNQ channels on membrane voltage (Figures 3D,E) and contraction (Figure 5E). Thus, we conclude that KCNQ channels have functional redundancy with BK channels, especially when combined with strong G_q/11_-mediated muscarinic inhibition, which minimizes their role during muscarinic-evoked contractions. Further, we can conclude that KCNQ channel inhibition is unlikely to underlie muscarinic depolarization of ASM. Indeed, the roles of KCNQ and BK channels may be considered complementary. Low-voltage threshold KCNQ channels likely control membrane potential at resting voltages when intracellular calcium is low. During muscarinic signaling, BK channels assume a greater role due to the depolarized membrane potential and elevated calcium that support BK channel activation.

The *in vitro* constriction study supports a supplementary role for KCNQ channels in control of airway contractility when BK K^+^ channel activation is perturbed. This is further supported by *in vivo* experiments showing that RTG reduces the PenH index. Although the whole animal plethysmography may also reflect changes in breathing independent of airway resistance (Glaab et al., 2007), the results are consistent with the constriction experiments. Thus, retigabine, a drug already approved for human treatment in epilepsy, might be an effective bronchodilator particularly under pathological conditions where BK channel expression or function is perturbed. Indeed, human polymorphisms for the BK channel β1 subunit have been identified that perturb BK/β1 function and correlate highly with increased asthma severity (Seibold et al., 2008) and retigabine in these individuals may serve as a tailored therapy. As well, there are a number of studies showing downregulation of BK channel β1 in diseases such as hypertension (Nieves-Cintron et al., 2008), aging (Nishimaru et al., 2004), diabetes (McGahon et al., 2007a,b; Zhang et al., 2010; Lu et al., 2012), and bladder overactivity (Chang et al., 2010). If BK channels are also inhibited during diseases of the airway such as asthma, then KCNQ channels may become particularly more relevant in control of airway contractility.

This study in rodents support past studies showing KCNQ channel expression in ASM of guinea pig and human tissues (Brueggemann et al., 2012). However, these studies contrast with previous studies insofar as KCNQ channel effects on airway contractility. Previous studies assayed diameter changes of bronchioles in human lung slices. In those studies, blockade of KCNQ channels profoundly enhanced histamine-induced constriction (Brueggemann et al., 2012). In these studies, we measured isometric contractions using tracheal rings and saw no significant effect of KCNQ blocker on membrane voltage or contractility during application of muscarinic agonists, the main physiological stimulus for ASM contraction in the living animal. We speculate that the discrepancy may be due to differences in the relative contribution of KCNQ channels in rodent and human lung. Rodents may have a greater contribution of BK or other K^+^ channel in lower ASM, whereas human lung may be more reliant on KCNQ channels. Further studies should elucidate the relative contribution of these two channel types in lower airway of rodents.

### Conflict of interest statement

The authors declare that the research was conducted in the absence of any commercial or financial relationships that could be construed as a potential conflict of interest.
